# Depressive Symptoms of Chinese Children: Prevalence and Correlated Factors among Subgroups

**DOI:** 10.3390/ijerph15020283

**Published:** 2018-02-07

**Authors:** Mi Zhou, Guangsheng Zhang, Scott Rozelle, Kaleigh Kenny, Hao Xue

**Affiliations:** 1 College of Economics and Management, Shenyang Agricultural University, Shenyang 110866, China; zhoumisyau@163.com; 2 School of Business, Liaoning University, Shenyang 110031, China; gshzhang@163.com; 3 Rural Education Action Program, Freeman Spogli Institute for International Studies, Stanford University, Stanford, CA 94305, USA; rozelle@stanford.edu (S.R.); krkenny@stanford.edu (K.K.); 4 School of Economics and Management, Northwest University, Xi’an 710069, China; 5 Center for Experimental Economics in Education, Shaanxi Normal University, Xi’an 710127, China

**Keywords:** childhood depression, China, depressive symptoms, left-behind children, migrant children

## Abstract

Economic growth and socioeconomic changes have transformed nearly every aspect of childhood in China, and many are worried by the increasing prevalence of mental health issues among children, particularly depression. To provide insight into the distribution of depressive symptoms among children in China and identify vulnerable groups, we use data from the 2012 China Family Panel Survey (CFPS), a survey that collected data from a large, nationally representative sample of the Chinese population. Using the CFPS data, we construct a sample of 2679 children aged 10–15 years old from 25 provinces in China. According to our results, the incidence of depression varies by geographic area. Specifically, we find that rates of depressive symptoms are significantly lower in urban areas (14% of sample children) than in rural areas (23% of sample children). Our results also show that children from ethnic minorities, from poorer families, and whose parents are depressed are more likely to be depressed than other children. In contrast, we find that depressive symptoms do not vary by gender.

## 1. Introduction

In recent decades, economic growth and socioeconomic changes have transformed nearly every aspect of childhood in China, especially education. Parents invest heavily in their children in the hopes that they will be able to thrive in China’s highly competitive education system [1]. In addition to these increased investments (and perhaps more importantly), it is believed that children are now able to access higher quality education than in years past, which in turn increases their human capital and improves overall life outcomes [2].

However, there is another societal trend that may undermine this progress. China has seen a worrying increase in the prevalence of mental health issues among children, particularly depression [3]. Unfortunately, there has been little research that uses recent data based on large, nationally-representative samples that have tried to quantify the magnitude of this problem. In 2000, a study using data from a single province found that one-third of sample children reported a history of depression [4]. Other studies show that the prevalence rate of depression among left-behind children is reported to range from 12.1% to 51.4% when using data from one or more provinces [5].

These high rates of childhood depression in China are especially concerning considering the degree to which depressive symptoms harm educational outcomes. Research has found that depressed children are more likely to perform poorly academically and have poorer outcomes later in life [6]. If depressive symptom rates are high across all of China, then the country may be suffering from a substantial human capital loss due to early-onset depression [7]. In addition, depressive symptoms in children have been found to be associated with criminal, anti-social, and other deviant behaviors [8,9]. Therefore, supporting the mental health of children could have positive societal impacts, such as reducing crime, raising earnings, and promoting health and education [10,11].

Fortunately, it appears that China’s government has become aware of this issue. One response was seen in 2012 when the Ministry of Education launched a program to provide qualified mental health education and services to students in urban primary and middle schools. The pilot program included guidelines such as reducing student workload, implementing mental health curriculums, establishing counseling offices, and improving student-teacher relationships [12]. Although the establishment of this program is encouraging, it fails to address childhood depression in rural China. In one of the only studies we could find on child depressive symptoms in rural China, which used data collected in only two counties, the measured rates of depressive symptoms were high [4]. Thus, while it seems from the few existing studies that depression rates are high in rural areas, there has been little research that systematically studies childhood depressive symptoms across all areas of China.

Future efforts to combat childhood depressive symptoms would also benefit from a better understanding of which sub-populations of children are more or less vulnerable to developing depression. This is especially true if resources (human and fiscal) are relatively scarce and need to be targeted in order to efficiently address the problem. Unfortunately, there have been almost no high-quality studies on how depressive symptoms vary among major sub-populations of Chinese children. Based on our reading of the literature, there are no studies using nationally representative data that report differences in the prevalence of childhood depressive symptoms between sub-populations, such as between rural and urban areas, between rich and poor families, between male and female children, or between Han and Non-Han ethnic minorities.

The overall goal of this paper is to examine childhood depressive symptoms in China today. However, the paper goes beyond looking at overall depressive symptom rates, as we not only examine the overall prevalence of depressive symptoms among children, but also seek to measure and compare the levels of depressive symptoms for key subgroups of children. The analysis uses a nationally representative dataset and an internationally recognized scale of depression (CES-D) to examine depression levels among urban and rural children aged 10–15 years old. The ultimate objective is to build a repository of information on depression among rural children in China, which can be used as a decision-making tool to help top leaders target their investments and create policies that will be aimed at improving the mental health of China’s most vulnerable children.

To meet the above objectives, the rest of the paper will be organized as follows: the next section of the paper describes the data and the selection of the sample. The following section presents results. The final section summarizes and draws conclusions.

## 2. Methods

### 2.1. Data and Sampling

The data used for this study come from the second wave of China Family Panel Studies (CFPS) conducted in 2012. The CFPS is a nationally representative, longitudinal social survey that was launched in 2010 and is conducted biennially by the Institute of Social Science Survey (ISSS) at Peking University. With a survey design based on the Panel Survey of Income Dynamics (PSID), the National Longitudinal Survey of Youth (NLSY), and the Health and Retirement Study (HRS) in the United States, the CFPS focuses on a range of topics related to educational outcomes, economic activities, migration, physical and mental health, and family dynamics. The survey collects data at three levels, including individual, family, and community levels.

The CFPS surveyed respondents in sampling units in 25 provinces (all provinces except Xinjiang, Tibet, Qinghai, Inner Mongolia, Ningxia, and Hainan), a sampling frame which represents 95% of the Chinese population. To generate a nationally and provincially representative sample, the CFPS adopted a “Probability-Proportional-to-Size” (PPS) sampling strategy with multistage stratification and carried out a three-stage sampling process. First, 162 county-level units were randomly selected across 25 provinces. Second, 640 village-level units (villages in rural areas and neighborhoods/communities in urban areas) were selected. In the third stage, 6317 households from the village-level units were selected according to the study’s systematic sampling protocol. All members of each household who were at home during the time of the survey were interviewed.

The 2012 CFPS survey originally included 3057 children aged 10 to 15 years old. We chose to evaluate children in this age range because they were old enough to fill out the survey form on their own. After excluding 378 observations that were missing more than four responses to the 20 items in the CES-D scale, we reduced our sample to 2679 children.

### 2.2. Measures

The key block of the survey questionnaire for this study was made up of questions from the Center for Epidemiologic Studies Depression Scale (CES-D). In addition, there were a number of smaller blocks that were used to enumerate student and family characteristics and other control variables. Specifically, the data allow us to generate variables that measure personal characteristics, including gender, ethnicity, and boarding status; and household characteristics, including poverty and parental depression status. We also asked a set of questions that enabled us to create a variable representing the living arrangements of children, including: “Where is your household currently registered?”, “Do you live in an urban or rural community?”, and “In the past year, how long did the child live with his/her father/mother?”

The CES-D scale has been used widely in the international literature to evaluate symptoms of depression [13,14]. The CES-D scale includes 20 items and is scored on a Likert scale with four possible answers corresponding to how often the respondents experienced a given emotion within the past week: “rarely or none of the time (less than 1 day)”, “some or a little of the time (1–2 days)”, “occasionally or a moderate amount of time (3–4 days)”, and “most or all of the time (5–7 days)”. Possible scores range from 0 to 60 and a score of 17 or higher is indicative of depression. In addition to determining whether or not children display depressive symptoms, we also used scores on the CES-D to determine the severity of a child’s depression. Specifically, we set three different levels of depression severity: a CES-D score between 17 and 23 indicates “mild depression,” a score between 24 and 28 indicates “moderate depression”, and scores of 29 or higher indicate “severe depression.” If more than four responses were missing from a single observation, then we did not include the observation in the sample [15]. The Chinese version of CES-D has been used in previous research [16] and its reliability and validity has been tested among Chinese populations [17]; therefore, the scale has been shown to be appropriate for use in China [18]. Because of this, we believe that this scale will not only allow us to examine the depression statuses of children in our sample, but also compare depressive symptoms among different sub-populations of children, such as children living in urban and rural areas of China.

To collect individual-level data on our sample children, we referred to the CFPS Children’s survey. For our analysis, the individual characteristics of students included gender, ethnicity (Han or non-Han minority), and whether the students board at school. Boarding status was only relevant, however, for rural students, as almost all urban students lived at home. Our analysis also included household characteristics such as household income and parental depression status. To evaluate household income, we used responses to the question, “During the 2011–2012 school year, what was the net income of your household?”, data from the rural poverty line (369 USD family net income per year in 2012), and the minimum cost of living in urban areas (1283 USD family net income per year in 2012). With this information, we divided children’s households into two groups: a (relatively) *rich group* and a (relatively) *poor group*. The group of rich households included 1601 rural households and 437 urban households, while the group of poor households included 389 rural households and 252 urban households. We also used parent responses from the 2012 CFPS survey to determine the depression status of parents, using the same CES-D questions that we used to evaluate children in our sample.

To examine the effect of living arrangements on the depressive symptoms of children, we used each child’s residency status (or hukou) and whether they lived with their parents or not (who might have out-migrated) to divide the sample into five subgroups. Among the five subgroups, two subgroups were “types” of urban children: urban-origin children (UC), who have urban hukous and reside in urban areas, and migrant children (MC), who have rural hukous but had resided in urban areas at least 6 months out of the previous year. We also created three “types” of rural children: left-behind children (LBC), who have rural hukous and resided in rural areas while both of their parents worked and lived outside of the household for at least 5 months out of the previous year; father-only migration children (LBCF), who have rural hukous and resided in rural areas with their mother while only their father worked and lived outside of the household for at least 5 months out of the previous year; and children living with both parents (CLP) in their rural communities. In our sample 76 children lived with their fathers while their mothers migrated for work. This is a relatively small number of children and this subgroup comprised only 2.8% of our sample. Because we believe that the sample size is too small to yield any meaningful insight into the situation of this subgroup of children, we did not include this subgroup in our analysis.

### 2.3. Statistical Analyses

For statistical analysis, we use Cronbach’s α for the whole sample to test the internal consistency. We compared the CES-D scores of different subgroups of children using *t*-tests. Specifically, we examined differences in CES-D scores between: Han Chinese and ethnic minority children; male and female children; boarding students and children living at home; richer and poorer students; and children whose parents do and do not have depression symptoms. Finally, we also use *t*-tests to examine differences in the mean CES-D scores of different subgroups of children based on residency status: urban-origin children (UC), migrant children (MC), left-behind children (LBC), father-only migration children (LBCF), and children living with both parents (CLP). All analysis was performed using Stata version 14 (Stata Corporation, College Station, TX, USA).

## 3. Results

### 3.1. Descriptive Statistics

The characteristics of our sample are displayed in Table 1. As can be seen from the data, 25.7% of children live in urban areas and 74.3% live in rural areas. Among the children living in urban areas, 26.7% are migrant children and 73.3% are urban-origin children. In our sample of rural children, 26.0% of children are LBCs, 13.5% are LBCFs, and 56.7% are CLPs. In terms of gender, 52.2% of the children in our sample are male and 47.9% are female. In terms of minority status, 87.5% of children were Han Chinese, while 12.5% were non-Han ethnic minorities. We also found that 76.4% of children in our sample lived at home and 23.6% of children boarded at school. In terms of household poverty status, we found that 23.9% of children in our sample were from (relatively) rich households and 76.1% were from (relatively) poor households. Also, 47.2% of children in our sample had a parent who displayed symptoms of depression, while 52.8% of children in our sample did not. The CES-D scores showed internal consistency, and Cronbach’s α for the whole sample was 0.809.

### 3.2. The Prevalence of Depressive Symptoms among Sample Children

Table 2 summarizes the severity of depression in our sample, categorized by CES-D scores. As can be seen in the table, 20.3% of sample children display depressive symptoms. However, the prevalence of depression is not evenly balanced between rural and urban areas. Specifically, 22.7% of rural children display symptoms of depression, while only 13.5% of urban children exhibit depressive symptoms, and this difference is significant at the 1% level. We also find that the severity of depressive symptoms varies between rural and urban areas. Rural children are more likely to suffer from mild (16.8% of rural children) or moderate depression (3.9% of rural children) than their urban peers (10.7% and 1.3% of urban children, respectively), and these differences are significant at the 1% level. Urban and rural children, however, are almost equally likely to suffer from severe depression (2.1% of rural vs. 1.5% of urban children); the difference is not significant.

### 3.3. Depressive Symptoms among Subgroups of Children

When we compare subgroups of children who differ by individual characteristics, we find that significant differences emerge between Han and ethnic minority children, but there are no significant differences in depression status based on gender or boarding status. Our results show that ethnic minority children are significantly more likely to be depressed than Han children in rural areas (significant at the 1% level—Table 3, Rows 1 and 2). Although the point estimates for the gender comparisons show that a higher share of female children are depressed than male children in both the urban and rural samples, the differences are not significant for either urban or rural children (Rows 3 and 4). Similarly, there is no significant difference between subgroups based on boarding status (Rows 5 and 6).

In terms of household-level characteristics, we find that both household income and parental depression are significantly correlated with childhood depression among children in our sample. The difference in average CES-D scores between the rich and the poor groups in both urban and rural areas suggest that children from poorer households are more likely to be depressed than those from richer households (significant at the 1% level—Table 3, Rows 7 and 8). Parental depression is also found to be significantly correlated with childhood depression in both urban and rural areas (Rows 9 and 10).

**Figure 1:**
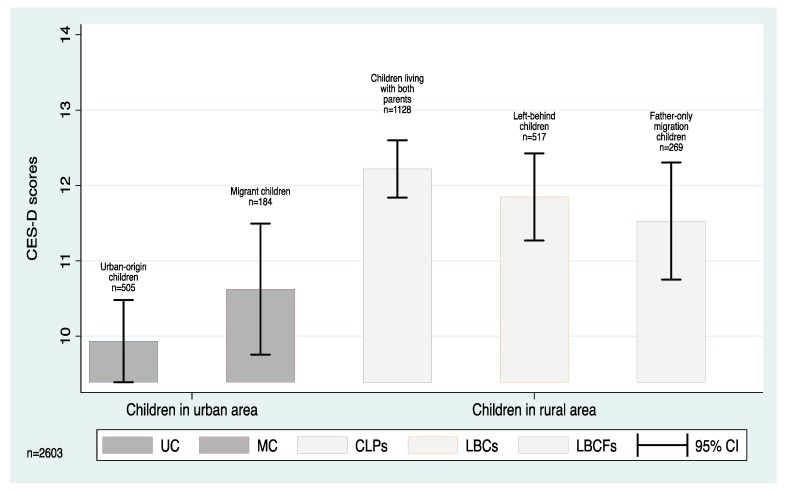
The Differences of CES-D Scores among Children of Different Household Migration Patterns and Residency Statuses with 95% Confidence Interval (CI). Source: CFPS (2012). Notes: Migrant children (MC) are specified as children under the age of 16 who have rural Hukous and migrate to urban areas for at least 6 months per year; Left-behind children (LBC) are specified as children under the age of 16 who have been left-behind in rural areas while both parents migrate to other areas for at least 5 months per year. We do not evaluate the sample of “Mother-only migration children”, because this sample size is too small. The *p*-value of the difference between Migrant children and Urban-origin children is 0.1951 by *t*-test. The *p*-value of the difference between Migrant children and all rural children is 0.0039 by *t*-test. The *p*-value of the difference between Migrant children and children living with both parents is 0.0019 by *t*-test. The *p*-value of the difference between Left-behind children and children living with both parents is 0.2866 by *t*-test. The *p*-value of the difference between Father-only migration children and children living with both parents is 0.1178 by *t*-test.

When comparing the two urban subgroups (UCs and MCs) we find little difference in average CES-D score. Specifically, we find that mean CES-D scores of urban children and migrant children are both about 10, with no statistically significant difference between the two (Figure 1). Similarly, we find no significant differences in average CES-D scores between the three rural subgroups (CLPs, LBCs, and LBCFs). In rural areas, we find that mean CES-D scores of CLPs, LBCs, and LBCFs are about 12.2, 11.9 and 11.5, respectively. Although there are slight differences in the point estimates among the different groups of children residing in rural areas (that is, CLPs, LBCs and LBCFs), the gaps are statistically insignificant.

While there is little variation in average standardized CES-D scores of children within urban and rural areas, we do find significant differences between children residing in urban areas and those in rural areas. The gap in CES-D scores between MCs and rural subgroups (including CLPs, LBCs, and LBCFs) is even significant at the 1% level. This suggests that even though MCs were born and previously lived in rural areas, they may benefit from the environment and resources available in urban areas.

## 4. Discussion

Using nationally representative data from the CFPS, we demonstrate that the prevalence of childhood depressive symptoms in China is high. Specifically, according to the CES-D scale, 20.3% of all children in China display symptoms of depression. These rates are high compared to similar studies using nationally representative samples from other developing countries [19]. These estimates also support previous studies in China that report a high prevalence of childhood depression—although previous analyses used data with much smaller sample sizes from more limited geographic areas [20,21]. Taken together, these findings indicate that childhood depressive symptoms are indeed an issue in China, and steps must be taken to improve the mental health condition of Chinese children.

Although it appears that all children in China are vulnerable to developing depression, our results also show that the risk is particularly acute for certain subgroups of children. For example, we find in this study that children living in rural China are statistically significantly more likely to be depressed than those living in urban areas. In addition, we discovered that in rural areas, non-Han minority children are significantly more likely to be depressed than Han children. These findings are important because they have allowed us to identify sub-populations of children in China that are particularly vulnerable to depression. With this knowledge, it will be possible to develop policies and programs targeting these groups of children that can decrease childhood depressive symptoms across China.

Although no previous studies have comprehensively examined the prevalence of depressive symptoms among subgroups of children in China, our findings are supported by the international literature. For example, a study conducted in Australia found that children living in rural areas are more likely to be depressed than those living in urban areas [22]. In addition, research conducted in Pakistan has found that the prevalence of depressive symptoms was higher in rural than urban sample areas [23]. Findings from other countries on depressive symptoms among cultural and ethnic minorities also support the findings of our research. Studies from the United States consistently find high depressive symptom rates among African-Americans and other ethnic minorities [24,25,26]. A study in India also found that members of lower castes suffer disproportionately from depression [27].

Interestingly, we found that there was no difference between the depression levels of male and female children in our sample. This stands in contrast to other studies conducted in China and other developing countries that have found that females are significantly more vulnerable to developing depressive symptoms than males [4]. However, evidence suggests that this finding could be related to the age of our sample. A previous study found that there are no gender differences in depressive symptom rates in children, but after the age of 15 girls have significantly higher rates of depressive symptoms than boys [28]. Although all children in China certainly need additional mental health support, these findings suggest girls may not need any additional support during this stage in life.

This study has a number of strengths. First, our sampling frame represents 95% of the Chinese population, and therefore can be considered nationally representative. Second, the large sample size of our dataset (*n* = 2679) gives our research a high degree of statistical power and considerable external validity. Third, the Institute of Social Science Survey (ISSS) of Peking University collected all data using a common sampling strategy. Last, this paper focuses on the depressive symptoms of not only children from rural areas, but also urban and migrant children. The comparison of different types of children can give us more evidence on the effect of family migration on children’s depression.

Even with its strengths, our study also suffers from several limitations. First, the CFPS only collects self-reported information from children aged 10–15 years, which limits our potential sample to children in this age group. Second, because information on children’s depressive symptoms was not collected in other waves of the survey, we are unable to compare children’s depressive symptoms across different time periods. Future research examining childhood depressive symptoms in China would benefit from focusing on these research areas.

From a policy perspective, our results suggest that there are relatively high rates of childhood depressive symptoms in rural China as compared to urban areas of the country. This is concerning because high rates of depressive symptoms can impede the human capital accumulation of rural children and contribute to income inequality over the long-term. If China’s government wants to raise levels of human capital, particularly in rural areas, it will need to make more comprehensive efforts to improve the mental health of all children. This can be done by implementing mental health programming that provides support for vulnerable groups of children in rural areas of the country. Recent research has found that implementing school counseling programs is an effective method for reducing learning anxiety among school children in urban China [29]. It may be possible to address childhood depressive symptoms by implementing similar programs in rural schools. In designing effective programs, it is also necessary to pay attention to the heterogeneity of Chinese children. Several studies have reported large urban and rural differences in socioeconomic conditions and regional inequalities in child malnutrition [30]. Following this reasoning, it is important that programs are designed to be sensitive to the environment in which they are implemented.

## 5. Conclusions

Using nationally representative data, this study revealed the rates of depressive symptoms among Chinese children are significantly lower in urban areas (14% of sample children) than in rural areas (23% of sample children). We also discovered that children from ethnic minority groups, from poorer families, and whose parents are depressed are more likely to display depressive symptoms than other children, but depressive symptoms do not vary by gender. We believe our results can help policy makers develop targeted programs for depressed children in the future. 

## Figures and Tables

**Table 1:** Descriptive statistics.

		Observations	Share (%)	Depression Score	
				Mean	S.D.
		(1)	(2)	(3)	(4)
(1)	Urban	689	25.72	10.12	6.19
(2)	Migrant children (MC)	184	26.71	11.65	6.59
(3)	Urban-origin children (UC)	505	73.29	10.65	6.02
(4)	Rural	1990	74.28	12.09	6.61
(5)	Left-behind children (LBC)	517	25.98	11.85	6.70
(6)	Only father migrates (LBCF)	269	13.52	11.52	6.51
(7)	Children living with both parents (CLP)	1128	56.68	12.22	6.51
(8)	Male	1397	52.15	11.44	6.54
(9)	Female	1282	47.85	11.73	6.58
(10)	Han ^a^	2343	87.46	11.21	6.46
(11)	Ethnic Minority	336	12.54	14.14	6.62
(12)	Boarding ^b^	632	23.59	11.47	6.08
(13)	Living at home	2047	76.41	11.65	6.70
(14)	Rich	2038	23.93	11.40	6.52
(15)	Poor ^c^	641	76.07	12.15	6.65
(16)	Parent does not have depressive symptoms	1571	52.85	10.39	6.02
(17)	Parent has depressive symptoms	1078	47.15	13.31	6.92

Source: CFPS (2012); Note: ^a^ Because few non-Han ethnic minority children live in urban areas (only a very small share of urban children are non-Han ethnic minority and most of those belong to either the Manchu or Korean ethnic groups, which are known to be comparatively well-off ethnic minorities), we only report differences between Han and non-Han children from rural areas. ^b^ Because only a small share of urban children board at school, we only report differences between boarding and non-boarding students from rural areas. ^c^ A household is considered “poor” if the net family income is below the rural poverty line (369 USD family net income per year in 2012) or lower than the minimum cost of living in urban areas (1283 USD family net income per year in 2012).

**Table 2:** The proportion of sample children with different levels of depression severity—CFPS (2012).

	Total	Urban	Rural	Difference
	(1)	(2)	(3)	(2,3)
All Depression ^a^	0.203	0.135	0.227	−0.092 ***
Mild Depression ^b^	0.153	0.107	0.168	−0.060 ***
Moderate Depression ^c^	0.032	0.013	0.039	−0.026 ***
Severe Depression ^d^	0.019	0.015	0.021	−0.006

Source: CFPS (2012). Note: The symbol *** means that the *p*-value is less than 0.01 (*p* < 0.01). ^a^ “All levels of depression” refers to overall CES-D scores greater than or equal to 16. ^b^ “Mild depression” refers to CES-D scores between 17 and 23. ^c^ “Moderate depression” refers to CES-D scores between 24 and 28. ^d^ “Severe depression” refers to CES-D scores greater than or equal to 29.

**Table 3:** Differences in CES-D scores among sub-populations of rural children and urban children—CFPS (2012).

		Urban		Rural	
		Mean	Difference	Mean	Difference
		(1)	(2)	(3)	(4)
(1)	Ethnic Minority	n.a.	n.a.	14.92	3.31 ***
(2)	Han ^a^	n.a.		11.61	
(3)	Female	10.49	0.71	12.02	−0.13
(4)	Male	9.78		12.15	
(5)	Boarding ^b^	n.a.	n.a.	11.61	−0.60
(6)	Living at home	n.a.		12.21	
(7)	Poor ^c^	10.96	1.33 ***	12.92	1.04 ***
(8)	Rich	9.63		11.88	
(9)	Parent with depressive symptom	11.98	2.58 ***	13.01	2.15 ***
(10)	Parent without depressive symptom	9.40		10.86	

Source: CFPS (2012). Notes: *** is *p* < 0.01. ^a^ Since few Non-Han ethnic minority children live in urban areas (only 7.26% of urban children are Non-Han minority), we only report differences between Han and non-Han children from rural areas. ^b^ Because almost no urban children board at schools (only 6.24% of urban children board at school), we only report differences between boarding and non-boarding students from rural areas. ^c^ Families are classified as poor if net family income is below the poverty line (369 USD family net income per year in 2012) in rural areas or lower than the minimum cost of living in urban areas (1283 USD family net income per year in 2012).
